# Marek’s Disease Virus Cluster 3 miRNAs Restrict Virus’ Early Cytolytic Replication and Pathogenesis

**DOI:** 10.3390/v12111317

**Published:** 2020-11-17

**Authors:** Yifei Liao, Guoqing Zhuang, Aijun Sun, Owais A. Khan, Blanca Lupiani, Sanjay M. Reddy

**Affiliations:** Department of Veterinary Pathobiology, College of Veterinary Medicine & Biomedical Sciences, Texas A&M University, College Station, TX 77843, USA; liao.yifei@tamu.edu (Y.L.); gqzhuang2008@163.com (G.Z.); sunaijun225@163.com (A.S.); owais.khan@tvmdl.tamu.edu (O.A.K.); blupiani@tamu.edu (B.L.)

**Keywords:** Marek’s disease virus, miRNA, replication, lymphoid organ atrophy, pathogenesis

## Abstract

Herpesvirus-encoded microRNAs (miRNAs) have been discovered in infected cells; however, lack of a suitable animal model has hampered functional analyses of viral miRNAs in vivo. Marek’s disease virus (MDV) (*Gallid alphaherpesvirus* 2, GaHV-2) genome contains 14 miRNA precursors, which encode 26 mature miRNAs, grouped into three clusters. In this study, the role of MDV-encoded cluster 3 miRNAs, also known as mdv1-miR-M8-M10, in pathogenesis was evaluated in chickens, the natural host of MDV. Our results show that deletion of cluster 3 miRNAs did not affect virus replication and plaque size in cell culture, but increased early cytolytic replication of MDV in chickens. We also observed that deletion of cluster 3 miRNAs resulted in significantly higher virus reactivation from peripheral blood lymphocytes. In addition, pathogenesis studies showed that deletion of cluster 3 miRNAs resulted in more severe atrophy of lymphoid organs and reduced mean death time, but did not affect the incidence of MDV-associated visceral tumors. We confirmed these results by generating a cluster 3 miRNA revertant virus in which the parental MDV phenotype was restored. To the best of our knowledge, our study provides the first evidence that MDV cluster 3 miRNAs play an important role in modulating MDV pathogenesis.

## 1. Introduction

MicroRNAs (miRNAs) are approximately 22 nucleotide-long, non-coding RNAs that have been identified in animals, plants, and viruses. miRNAs associate with complementary sites in target mRNAs to regulate their post-transcriptional processes by inducing mRNA degradation and translation inhibition [1]. Numerous studies have revealed the importance of miRNAs in virus infection [2,3,4]. Most herpesviruses, including herpes simplex virus (HSV), Epstein–Barr virus (EBV), Kaposi’s sarcoma-associated virus (KSHV), and Marek’s disease virus (MDV), encode miRNAs, indicating the important role of miRNAs in diverse hosts [5,6,7,8]. Recently, EBV- and KSHV-encoded miRNAs have been shown to be important for viral transformation and oncogenesis [9,10,11]. However, the lack of suitable natural infectious animal models has limited the direct functional analyses of viral miRNAs in vivo.

MDV, an avian oncogenic alphaherpesvirus that causes a highly contagious neoplastic disease in chickens, is an ideal infectious model for herpesvirus related cancer research. Marek’s disease (MD) is an immunosuppressive disease in chickens, characterized by infiltration of T lymphoblastoid tumor cells in visceral organs and peripheral nerves. Upon infection in susceptible chickens, four phases of MDV-induced pathogenesis are generally recognized, including early cytolytic infection, latency, reactivation, and transformation [12]. Early cytolytic infection occurs primarily in B cells 3–6 days post-infection, and latency is established 7–10 days post-infection in activated CD4^+^ T lymphocytes. Reactivation describes the recurrence of cytolytic replication from latently infected T lymphocytes in response to intra- or extra-cellular stimuli. Transformation usually takes place in latently infected cells after 14 days post-infection [12]. The MDV genome encodes more than 100 genes; among them, *meq* (MDV EcoRI Q) [13], *pp38* (phosphorylated protein 38KD) [14], *pp14* [15], *vLIP* (viral lipase) [16], *RLORF4* [17], *vIL-8* (virus-encoded interleukin 8) [18,19], and *vTR* (virus-encoded telomerase RNA subunit) [20,21] play varying but significant roles in MDV pathogenesis and oncogenesis. An additional type of regulatory molecule, MDV-encoded miRNAs, was identified in both infected chicken embryonic fibroblasts and lymphoblastoid tumor cell lines [22]. To date, the presence of 26 mature miRNAs derived from 14 precursor miRNAs, assembled in three distinct miRNA clusters in the MDV genome, have been identified [22]. Cluster 1 miRNAs, or mdv1-miR-M9–M4, are located upstream of *meq*, and include six precursor miRNAs; they have been shown to be important for MDV oncogenicity. In particular, miR-M4, an ortholog of chicken miR-155, has been shown to play the major role in regulating the oncogenicity of MDV [23]. Cluster 2 miRNAs, or mdv1-miR-M11–M1, are located downstream of *meq*, and include three precursor miRNAs. Cluster 3 miRNAs, or mdv1-miR-M8–M10, are found within the 5′ end of MDV *LAT* latency-associated transcript), and include five precursor miRNAs [23]. The MDV immediate–early genes *ICP4* and *ICP27* have been identified as targets of mdv1-miR-M7-5p, a member of the cluster 3 miRNAs [24]. By targeting these two genes, mdv-miR-M7-5p may contribute to MDV pathogenesis.

To directly study the role of cluster 3 miRNAs in MDV pathogenesis, we generated deletion and revertant viruses using an MDV BAC (bacterial artificial chromosome) clone derived from the very virulent plus strain 686 [25]. Our results show that the deletion of cluster 3 miRNAs did not affect virus replication and plaque size in cell culture, suggesting that cluster 3 miRNAs are dispensable for virus growth in vitro. Using chickens as the viral host model, we demonstrate that cluster 3 miRNAs are actively involved in MDV early cytolytic infection and may contribute to reactivation, and also play a role in limiting MDV pathogenesis. Our study provides evidence that viral cluster 3 miRNAs modulate MDV replication and pathogenesis.

## 2. Materials and Methods

### 2.1. Cells

Chicken embryonic fibroblasts (CEFs) were cultured at 37 °C in Leibowitz–McCoy medium supplemented with 5% bovine calf serum and penicillin–streptomycin, in the presence of 5% CO_2_. CEFs were used for virus propagation, titration, virus reactivation assay, and DNA transfection for virus recovery.

### 2.2. Construction of Cluster 3 miRNA Deletion and Revertant Viruses

To construct cluster 3 miRNAs’ deletion and revertant viruses, we followed the two-step, Red-mediated recombination procedure as previously described, using a 686BAC clone generated from a very virulent plus 686 strain of MDV [25,26]. All related primers are listed in Table 1.

Briefly, the *Kan^R^*–I–SecI cassette was amplified from pEPkan-S, using cluster 3 *Kan^R^*-forward (F) and *Kan^R^*-reverse (R) primers with homologous sequences necessary for subsequent recombination events. The purified PCR product was electroporated into the 686BAC-containing *E. coli*, followed by the addition of arabinose to induce *I-Sec* expression, in order to generate single copy deletion mutants of cluster 3 miRNAs. The same procedure was repeated to delete the second copy of cluster 3 miRNAs, in order to generate the 686BAC ∆miR-C3 (miR = miRNA) deletion mutant. This double-deletion mutant was then used as backbone to generate a revertant virus, 686BAC ∆miR-C3-Re, through the reinsertion of cluster 3 miRNA sequences. All deletion and revertant BACs were screened by PCR using flanking primers (cluster 3-F and cluster 3-R), followed by DNA sequencing, and were analyzed by restriction fragment length polymorphism (RFLP). BAC DNAs were transfected into CEF to produce recombinant viruses.

### 2.3. In Vitro Growth Kinetics

In vitro growth kinetics of parental 686BAC, mutant 686BAC ∆miR-C3, and revertant 686BAC ∆miR-C3-Re viruses were determined as described previously [14]. Briefly, CEFs seeded on 60 mm plate were infected with 100 plaque-forming units (PFUs) of either virus. On days 1, 2, 3, 4, and 5 post-infection, infected cells were trypsinized, 10-fold serial dilutions were co-cultured with fresh CEFs seeded on 35 mm plates, and plaques at each dilution were counted at 7 days post-infection.

### 2.4. Indirect Immunofluorescence Assay (IFA) and MDV Plaque Size Measurement

To visualize the plaques of recombinant viruses and determine their size, an indirect immunofluorescence assay (IFA) was performed with infected CEFs. At 6 days post-infection, infected CEFs were washed with phosphate-buffered saline (PBS) and fixed with ice-cold acetone/methanol (6:4) for 10 min. After blocking with 5% non-fat milk, cells were incubated with the MDV pp38 monoclonal antibody H19 for 1 h, followed by another hour of incubation with goat anti-mouse FITC-labeled secondary antibody (Thermo Fisher Scientific, Waltham, MA, USA) at room temperature. Cells were then washed three times with PBS, and plaque images were obtained using a fluorescence microscope. Plaque areas were measured from 30 randomly selected plaques of each virus using Image J software, and are presented as average plaque area.

### 2.5. Immunohistochemistry (IHC) Assay

To study the replication of parental 686BAC, mutant 686BAC ∆miR-C3, and revertant 686BAC ∆miR-C3-Re viruses in chickens, the spleen was collected at 5 days post-inoculation (dpi), embedded in optimal cutting temperature (OCT) compound (Tissue-Tek, Sakura Finetek, Torrance, CA, USA), and frozen immediately in liquid nitrogen. Six to eight µm-thick cryostat sections were prepared and fixed with cold acetone for 10 min. Immunostaining was performed with MDV pp38 monoclonal antibody H19 and the Vectastain ABC kit (Vector Laboratories, Burlingame, CA, USA), according to the manufacturer’s instructions.

### 2.6. Nucleic Acid Isolation and Quantitative Polymerase Chain Reaction (qPCR)

Genomic DNA was isolated from virus-infected CEFs or chicken splenocytes using phenol–chloroform extraction protocol, and quantification of the MDV genome copy number was accomplished by qPCR, with primers targeting MDV-infected cell protein 4 (*ICP4*) and chicken glyceraldehyde 3-phosphate dehydrogenase (*GAPDH*), as described previously [17,27]. Results were presented as MDV genome copies per million splenocytes, with error bars representing standard error of the mean (SEM).

### 2.7. Gene Expression Analysis

Genomic DNA and RNA were isolated using TRIzol reagent (Thermo Fisher Scientific, Waltham, MA, USA), as per the manufacturer’s instructions, and cDNA synthesis was carried out as described previously [27]. qPCR was performed to determine the expression level of MDV cluster 3 miRNAs, which were normalized to viral genome copy numbers (determined as stated above) and presented as the relative abundance, with error bars representing SEM.

All qPCR assays were performed in a Bio-Rad iCycler iQ Multicolor Real-Time Detection System, using iTag SYBR Green Mastermix (Bio-Rad, Hercules, CA, USA).

### 2.8. Virus Reactivation Assay

To examine the reactivation of recombinant viruses, five chickens from each experimental group were randomly selected and bled on day 14 dpi for the virus reactivation assay. Briefly, buffy coats were obtained from heparinized blood by centrifugation at 500× *g* for 5 min. Lymphocytes were diluted to 10^6^ cells per ml and co-cultured with fresh CEFs seeded on 35 mm plates in duplicate, and plaques were counted 7 days later.

### 2.9. Evaluation of 686BAC ∆miR-C3 Pathogenesis in Chickens

One-day-old, specific pathogen-free (SPF) chickens (Charles Rivers Laboratories, Wilmington, MA, USA) were wing-banded at hatch and randomly sorted into experimental groups. The chickens were inoculated subcutaneously with 2000 PFUs of 686BAC ∆miR-C3, 686BAC ∆miR-C3-Re, or parental 686BAC. One group remained uninoculated and served as the negative control. Animal experiments were performed according to the approved protocol of the Texas A&M University Institutional Animal Care and Use Committee (IACUC).

### 2.10. Lymphoid Organ Atrophy

To evaluate the effect of the miR-C3 deletion in lymphoid organ atrophy, five chickens from each group were euthanized at 14 dpi, and their lymphoid organs (thymus and bursa) and body weights were measured. Lymphoid organ atrophy was calculated as the ratio of lymphoid organ weight to body weight multiplied by 100.

### 2.11. Pathogenesis Study

To compare the pathogenic properties of 686BAC ∆miR-C3, 686BAC ∆miR-C3-Re, and parental 686BAC viruses, the daily mortality of each group was recorded for 8 weeks. Chickens that died during the course of the experiment or were euthanized at the time of termination were necropsied and examined for MDV-specific gross visceral tumors.

### 2.12. Data and Statistical Analysis

For the MDV genome copy number, gene expression, virus reactivation assay, and lymphoid organ atrophy, results were presented as the mean of the assay, with error bars showing the SEM, and data were analyzed with the Student’s *t*-test. The mortality rate (presented as the percentage of survival at each dpi) of each group was plotted, and the trends of the survival curve were examined with log–rank and Wilcoxon tests. All statistical analyses were performed using GraphPad Prism (Version 5.01, GraphPad Software, Inc. La Jolla, CA, USA).

## 3. Results

### 3.1. Construction and In Vitro Characterization of Cluster 3 miRNA Deletion and Revertant Viruses

**Figure 1:**
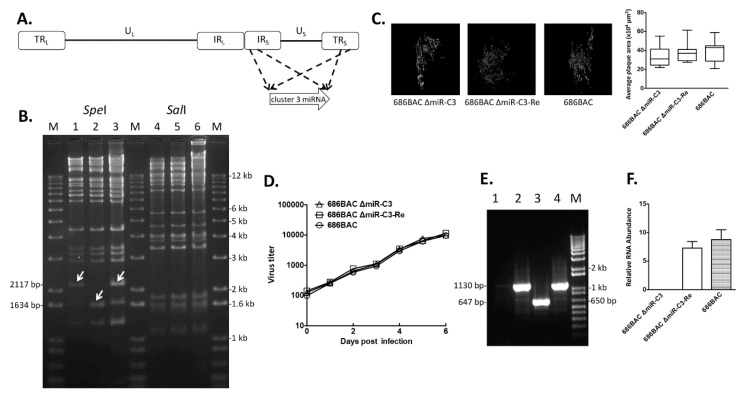
In vitro characterization of cluster 3 miRNA deletion and revertant viruses. (**A**) Marek’s disease virus (MDV) genome consists of unique long (U_L_) and short (U_S_) regions, each flanked by terminal and internal inverted repeat regions. Cluster 3 miRNAs are located within the internal (IR_S_) and terminal (TR_S_) repeat short regions of the MDV genome. (**B**) Restriction fragment length polymorphism (RFLP) analysis of bacterial artificial chromosome (BAC) DNA. Parental 686BAC (lanes 1 and 4), mutant 686BAC ΔmiR-C3 (lanes 2 and 5), and revertant 686BAC ΔmiR-C3-Re (lanes 3 and 6) DNA was digested with *Spe*I or *Sal*I. Arrows represent the DNA fragment in 686BAC ΔmiR-C3 that is different from the parental and revertant BACs. M: 1 kb plus DNA ladder. (**C**) Plaque area measurement. Chicken embryonic fibroblasts (CEFs) were infected with the indicated virus. Six days later, infected CEFs were fixed and subjected to an immunofluorescence assay with MDV pp38 antibody and Fluorescein isothiocyanate (FITC)-conjugated secondary antibody. For each virus, plaques were imaged (left), and the average plaque area of 30 randomly selected plaques is measured in µm^2^ (right). (**D**) In vitro growth kinetics. CEFs were infected with 100 plaque-forming units (PFUs) of parental 686BAC, mutant 686BAC ΔmiR-C3, or revertant 686BAC ΔmiR-C3-Re. At 1, 2, 3, 4, 5, and 6 days post-infection, infected cells were trypsinized, serially diluted, and seeded on fresh CEFs. Plaques were counted at 7 days post-infection and presented as virus titers. (**E**) PCR analysis of the MDV genome. Genomic DNA isolated from uninfected CEFs (lane 1) or parental 686BAC-infected (lane 2), mutant 686BAC ΔmiR-C3-infected (Lane 3), or revertant 686BAC ΔmiR-C3-Re-infected (Lane 4) CEFs were subjected PCR using cluster 3 miRNA flanking primers. M: 1 kb plus DNA ladder. (**F**) qPCR analysis of cluster 3 miRNA expression in vitro. Total RNA isolated from parental 686BAC-, mutant 686BAC ΔmiR-C3-, or revertant 686BAC ΔmiR-C3-Re-infected CEFs were subjected to complementary DNA (cDNA) synthesis, followed by qPCR analysis of cluster 3 miRNA expression. Cluster 3 miRNAs expression levels were normalized to viral genome levels and presented as relative RNA abundance.

To study the role of MDV cluster 3 miRNAs (Figure 1A) in MDV replication and pathogenesis, cluster 3 miRNA deletion (686BAC ∆miR-C3) and revertant (686BAC ∆miR-C3-Re) viruses were generated using two-step, Red-mediated recombination [26]. To confirm the integrity of 686BAC ∆miR-C3 and 686BAC ∆miR-C3-Re DNA, restriction fragment length polymorphism (RFLP) analysis was performed with *Spe*I and *Sal*I enzymes. As expected, digestion with *Spe*I showed that a 1634 bp-long fragment was generated from 686BAC ∆miR-C3 DNA (Figure 1B, lane 2), compared to a 2117 bp-long fragment from parental (Figure 1B, lane 1) and revertant BAC DNA (Figure 1B, lane 3), indicating that a 483 bp-long cluster 3 miRNA was successfully deleted in the 686BAC ∆miR-C3 BAC DNA. However, as cluster 3 miRNAs fall within the 16 kb-long fragments of *Sal*I digestion products, which cannot be resolved in 1% agarose gel, no differences in *Sal*I digestion fragments were observed among parental, deletion, and revertant BAC DNA (Figure 1B, lanes 4–6). The deletion of cluster 3 miRNAs in recombinant virus was further confirmed in infected chicken embryonic fibroblasts (CEF). A polymerase chain reaction (PCR) assay was performed using primers flanking cluster 3 miRNAs (Table 1), with genomic DNA extracted from 686BAC ∆miR-C3, 686BAC ∆miR-C3-Re, or parental 686BAC virus-infected CEFs. Results showed that an amplicon of 647 bp was generated from the 686BAC ∆miR-C3 genome (Figure 1E, lane 3), compared to 1130 bp from parental or revertant (Figure 1E, lane 2 or 4) virus genomes. In addition, as expected, the qPCR results from cDNA showed that the 686BAC ∆miR-C3 virus failed to produce cluster 3 miRNAs in infected CEFs (Figure 1F).

To determine the role of cluster 3 miRNAs in MDV replication in vitro, we examined both plaque size and growth kinetics of cluster 3 miRNA deletion and revertant viruses in the CEFs. At 6 days post-infection, no significant differences in average plaque area were observed among all three viruses (Figure 1C). In addition, the cluster 3 miRNA deletion virus grew similarly to tge parental and revertant viruses in CEFs (Figure 1D), suggesting that cluster 3 miRNAs are dispensable for virus growth in vitro.

### 3.2. Deletion of Cluster 3 miRNAs Increases Viral Load during Early Cytolytic Phase

**Figure 2:**
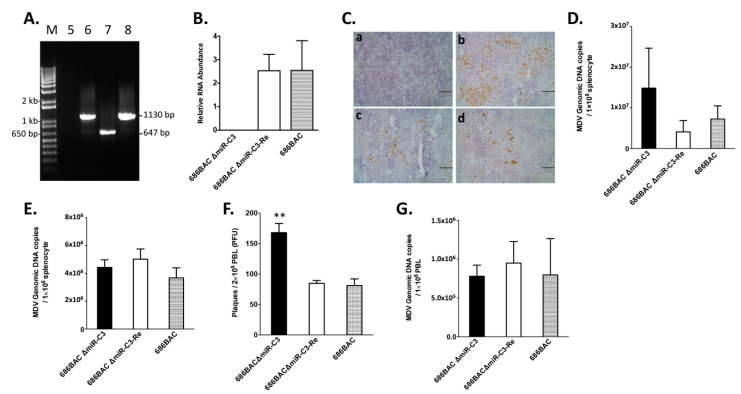
In vivo characterization of cluster 3 miRNA deletion and revertant viruses. One-day-old, specific pathogen-free chickens were inoculated with 2000 plaque-forming units (PFUs) of parental 686BAC, mutant 686BAC ∆miR-C3, or revertant 686BAC ∆miR-C3-Re. One group of chickens was not inoculated and served as a negative control. (**A**) PCR analysis of parental 686BAC, mutant 686BAC ∆miR-C3, and revertant 686BAC ∆miR-C3-Re genome. At 5 days post-inoculation (dpi), genomic DNA isolated from the peripheral blood lymphocytes (PBLs) of uninoculated or virus-infected chickens were subjected to PCR using cluster 3 miRNA flanking primers. M: 1 kb plus DNA ladder. (**B**) qPCR analysis of cluster 3 miRNA expression in vivo. Total RNA isolated from the splenocytes of parental 686BAC, mutant 686BAC ΔmiR-C3, or revertant 686BAC ΔmiR-C3-Re-inoculated chickens at 5 dpi was subjected to cDNA synthesis, followed by qPCR analysis of cluster 3 miRNA expression. Cluster 3 miRNA expression levels were normalized to viral genome levels, and are presented as the mean values of five chickens, with error bars representing the standard error of the mean (SEM). (**C**) Immunohistochemistry of spleens from uninoculated (a), mutant 686BAC ΔmiR-C3-inoculated (b), revertant 686BAC ΔmiR-C3-Re-inoculated (c), or parental 686BAC-inoculated (d) chickens at 5 dpi. All images were taken under the same magnification (scale bar = 100 µm). (**D**,**E**) MDV genome copy numbers in the spleen. Splenocytes from five chickens inoculated with mutant 686BAC ΔmiR-C3, revertant 686BAC ΔmiR-C3-Re, or parental 686BAC at 5 dpi (**D**) and 14 dpi (**E**) were subjected to genomic DNA isolation and qPCR analysis of the MDV genome copy number. Results are presented as average MDV genomic DNA copies per 1 × 10^6^ splenocytes, with the error bar representing SEM. (**F**,**G**) PBLs isolated from five chickens inoculated with mutant 686BAC ΔmiR-C3, revertant 686BAC ΔmiR-C3-Re, or parental 686BAC at 14 dpi were subjected to a virus reactivation assay (**F**) or MDV genome copy number of the PBLs (**G**). Results are presented as the mean value, with the error bar representing SEM. ** *p* < 0.01.

To determine the role of cluster 3 miRNAs in MDV replication in vivo, specific pathogen-free chickens were inoculated with 2000 PFUs of 686BAC ∆miR-C3, 686BAC ∆miR-C3-Re, or 686BAC virus. One group of chickens were not inoculated and served as the negative control. Similar to the in vitro results, deletion of cluster 3 miRNAs from the 686BAC ∆miR-C3 virus genome was confirmed in inoculated chickens (Figure 2A), and no expression of cluster 3 miRNAs was detected in the spleen of 686BAC ∆miR-C3 virus-inoculated chickens (Figure 2B). Interestingly, we found that at 5 days post-inoculation (dpi), there were more cells expressing MDV pp38 in the spleens of chickens inoculated with 686BAC ∆miR-C3 (Figure 2Cb) compared to revertant (Figure 2Cc) or parental (Figure 2Cd) virus-inoculated chickens. We also analyzed MDV genome copy numbers in the splenocytes of chickens inoculated with 686BAC ∆miR-C3, 686BAC ∆miR-C3-Re, or 686BAC virus at 5 dpi. As shown in Figure 2D, an increase (albeit not statistically significant) of MDV genome copy number was observed in the splenocytes of 686BAC ∆miR-C3 virus-inoculated chickens, compared to revertant and parental virus-inoculated chickens. However, the 686BAC ∆miR-C3 virus load was similar to revertant and parental viruses at 14 dpi (Figure 2E). Overall, these results suggest that cluster 3 miRNA deletion resulted in an increased viral load during early cytolytic phase.

### 3.3. Deletion of Cluster 3 miRNAs Significantly Increases Virus Reactivation from Peripheral Blood Lymphocytes (PBL)

MDV reactivation results in a switch from latency to cytolytic infection. To study the role of cluster 3 miRNAs in virus reactivation, peripheral blood lymphocytes (PBLs) were isolated from chickens at 14 dpi and subjected to a virus reactivation assay by co-culturing with a fresh CEF. Compared to revertant and parental viruses, significantly more virus was recovered in CEF co-cultured with PBL isolated from 686BAC ∆miR-C3 virus-inoculated chickens (Figure 2F); however, the levels of MDV genome copy numbers in PBLs isolated from all three types of virus-inoculated chickens were similar (Figure 2G). These results suggest that cluster 3 miRNAs may play a role in virus reactivation, but not the establishment of latency.

### 3.4. Deletion of Cluster 3 miRNAs Modulates MDV Pathogenesis

**Figure 3:**
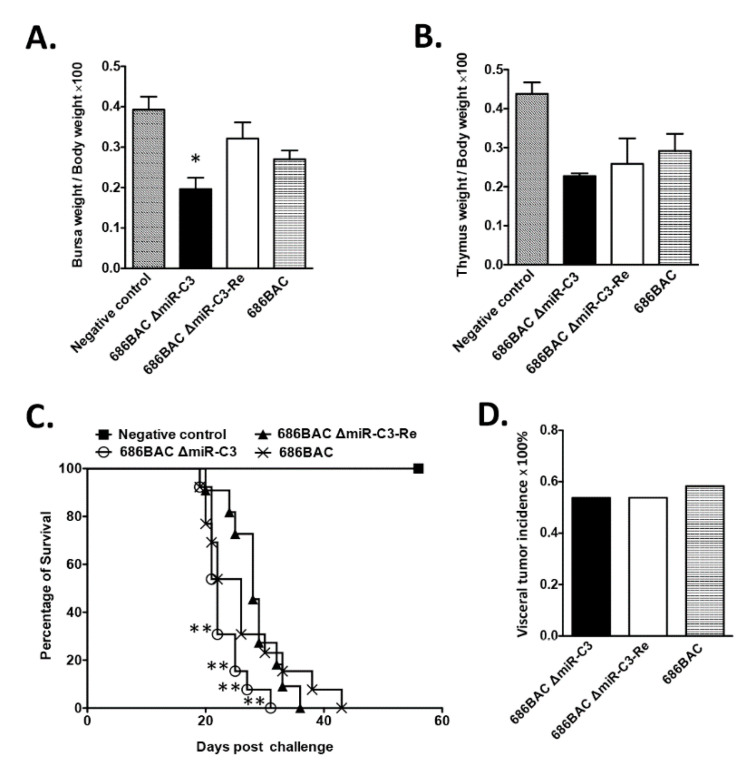
Evaluation of mutant 686BAC ΔmiR-C3, revertant 686BAC ΔmiR-C3-Re, or parental 686BAC pathogenesis in chickens. One-day-old, specific pathogen-free chickens were inoculated with 2000 plaque-forming units (PFUs) of parental 686BAC, mutant 686BAC ∆miR-C3, or revertant 686BAC ∆miR-C3-Re. One group of chickens remained inoculated and served as a negative control. (**A**,**B**) Lymphoid organ atrophy. At 14 days post-inoculation (dpi), body and lymphoid organs (bursa and thymus) weights of the negative control and inoculated chickens were measured. Results are presented as the average ratios of (bursa to body weight) × 100 (**A**) and (thymus to body weight) × 100 (**B**), with the error bar representing the standard error of the mean (SEM). (**C**,**D**) Pathogenesis of recombinant viruses. Inoculated and negative control chickens were maintained for 8 weeks. The mortality rate (presented as percentage of survival at each dpi) of each group is plotted, and the trends of the survival curve were examined with log–rank and Wilcoxon tests (**C**). Visceral tumor incidence in each experimental group (**D**). * *p* < 0.05, ** *p* < 0.01.

To study the role of cluster 3 miRNAs in MDV pathogenesis, one-day-old chickens were inoculated with 2000 PFUs of 686BAC ∆miR-C3, 686BAC ∆miR-C3-Re, or 686BAC virus, and uninoculated chickens were used as a negative control. It has been described that early cytolytic infection with highly pathogenic MDV causes lymphoid organ atrophy in chickens [28]. At 14 dpi, the bursa, thymus, and body weights of five chickens from each group were measured. Our results showed that the 686BAC ∆miR-C3 virus induced more severe lymphoid organ atrophy than the parental and revertant viruses in inoculated chickens (Figure 3A,B), especially in the bursa (Figure 3A), where the difference was significant. In addition, we observed that inoculation of 686BAC ∆miR-C3 virus resulted in a significantly shorter mean death time (22 days) in chickens, compared to revertant- (28 days) and parental (26 days)-inoculated chickens (Figure 3C). However, no differences were observed for the incidence of MDV-associated visceral tumors among all three groups (Figure 3D). Taken together, these results suggest that the deletion of cluster 3 miRNAs shortened the mean death time, but did not affect the tumor incidence in inoculated chickens.

## 4. Discussion

MDV is a highly contagious and oncogenic alphaherpesvirus that induces high mortality in chickens. Although MDV has been successfully controlled by vaccinations, it continues to evolve and causes disease outbreaks in vaccinated flocks. These highly virulent strains cause increased early cytolytic infection that results in severe lymphoid organ atrophy, immunosuppression, and neurological disease [29,30,31]. In recent years, three clusters of miRNAs have been identified within the repeat regions of MDV genome; in particular, miR-M4 within the cluster 1 miRNAs has been shown to be important for MDV oncogenicity [23]. Although mdv1-miR-M7-5p, a member of the cluster 3 miRNAs, was shown to target MDV *ICP4* and *ICP27* genes, the importance of cluster 3 miRNAs in MDV pathogenesis has not yet been studied [24].

In the present study, we examined the role of the cluster 3 miRNAs in MDV replication and pathogenesis by generating deletion and revertant mutant viruses. As cluster 3 miRNAs map to the newly identified first intron of MDV latency-associated transcripts (LATs), a cluster of non-coding spliced RNAs [24], we speculated that the deletion of cluster 3 miRNAs should not affect the expression and function of LATs. Our results show that the deletion of cluster 3 miRNAs did not affect the plaque size and growth kinetics of MDV in cell culture (Figure 1), suggesting that it is not essential for virus growth in vitro. However, we found that deletion of cluster 3 miRNAs enhanced MDV early cytolytic replication in the spleen, as shown by more cells expressing MDV pp38 and a higher viral genome copy number in 686BAC ∆miR-C3-inoculated chickens during early cytolytic phase (5 dpi), but did not affect the viral load at 14 dpi (Figure 2C–E). Early studies have shown that the regulation of early cytolytic infection correlates with incidence of lymphomas [14,17,19,32]. Even though we did not observe a higher incidence of MDV-associated tumors in 686BAC ∆miR-C3-inoculated chickens, the deletion of cluster 3 miRNAs resulted in significantly more severe bursa atrophy and a shorter mean death time for the chickens (Figure 3), both of which are indicators of highly virulent MDV. These data suggest that cluster 3 miRNAs may play distinct regulatory roles in controlling MDV early cytolytic replication and pathogenesis, leading to a less severe infection pattern to achieve life-long infection.

We also observed that inoculation with 686BAC ∆miR-C3 increased plaque recovery from PBLs at 14 dpi, without affecting the MDV genome copy number (Figure 2F,G). Our data provide evidence that MDV cluster 3 miRNAs may be involved in regulating MDV reactivation, the regulatory mechanisms of which will need further study. Herpesvirus reactivation is a complex process that can be induced by internal and external stimuli, including viral and cellular factors, as well as chemicals [33]. MDV cluster 3 miRNA may target viral or cellular factors to regulate MDV replication and reactivation, as one member of cluster 3 miRNAs, mdv1-miR-M7-5p, has been proven to repress the expression of two MDV immediate–early genes, *ICP4* and *ICP27* [24]. Recently, KSHV-encoded miR-K9 was identified as a “finely-tuned” switch of viral reactivation by its targeting of the major lytic switch protein RTA [34], and KSHV miR-K5 was shown to facilitate reactivation by targeting BCL2-associated transcription factor 1 (BCLAF1) [35]. These results suggest that herpesvirus miRNA-mediated regulation may afford viruses the ability to adjust their infectious strategies in response to environmental changes. Taken together, our results point out the role of cluster 3 miRNAs in MDV replication and pathogenesis, although the molecular mechanisms behind them remain to be studied. Considering the fact that cluster 3 miRNAs contain five precursor miRNAs (mdv1-miR-M8, mdv1-miR-M13, mdv1-miR-M6, mdv1-miR-M7, and mdv1-miR-M10) [23], future studies will be needed to precisely analyze the specific functions of individual miRNA precursors.

## 5. Conclusions

In summary, our study uncovered the role of cluster 3 miRNAs in MDV replication and pathogenesis by generating deletion and revertant viruses. We demonstrated that cluster 3 miRNAs are not critical for MDV replication in vitro and tumorigenesis in chickens; however, it is important to modulate MDV early cytolytic replication and pathogenesis in chickens. Thus, our study provides a novel insight of herpesvirus miRNAs in the regulation of viral replication and pathogenesis in its natural host.

## Figures and Tables

**Table 1:** List of primers used in the construction of cluster 3 microRNA (miRNA) deletion viruses.

Primer Name	Sequence (5′ to 3′)	Purpose
Cluster 3 *Kan^R^*-F	**GGAATAAACGTTGTGATACGCGATCGAGTTTTCGTGGCATATTCCTACGG** AGGATGACGACGATAAGTAGGG	Forward primer for amplification of *Kan^R^* cassette gene with MDV sequences flanking MDV cluster 3 miRNAs
Cluster 3 *Kan^R^*-R	**CGTTTACTTCCTAAGTCATCGCTCTTTAGTTGGGAGGAAAGTTTCCTAGACCGTAGGAATATGCCACGAAAACTCGATCGCGTATCACAACGTTTATTCC** CAACCAATTAACCAATTCTGATTAG	Reverse primer for amplification of *Kan^R^* cassette gene with MDV sequences flanking MDV cluster 3 miRNAs
Cluster 3-F	GTCCTCGTTGAATAGATGA	Amplification for MDV cluster 3 miRNAs with its flanking sequences of MDV
Cluster 3-R	AGGGGCGCATATACAGTC	

F: forward primer; R: reverse primer; MDV: Marek’s disease virus. Underlined sequences indicate the sequences from plasmid pEPkan-S used to amplify the *Kan^R^* gene cassette. Sequences in bold indicate MDV genome sequences flanking the MDV cluster 3 miRNAs.
